# A newly developed tetraplex real‐time RT‐PCR for simultaneous screening of influenza virus types A, B, C and D

**DOI:** 10.1111/irv.12613

**Published:** 2018-10-22

**Authors:** Dinah Henritzi, Bernd Hoffmann, Silke Wacheck, Stefan Pesch, Georg Herrler, Martin Beer, Timm C. Harder

**Affiliations:** ^1^ Institute of Diagnostic Virology Friedrich‐Loeffler‐Institut (FLI) Greifswald‐Insel Riems Germany; ^2^ IDT Biologika GmbH Dessau‐Rosslau Germany; ^3^ University of Veterinary Medicine Hannover, Foundation Hannover Germany

**Keywords:** European surveillance, influenza virus types A, B, C and D, multiplex RT‐qPCR, swine

## Abstract

**Background:**

Human‐ or avian‐to‐swine transmissions have founded several autonomously circulating influenza A virus (IAV) lineages in swine populations that cause economically important respiratory disease. Little is known on other human influenza virus types, like B (IBV) and C (ICV) in European swine, and of the recently detected novel animal influenza virus type D (IDV).

**Objectives:**

Development of a cost‐effective diagnostic tool for large‐scale surveillance programmes targeting all four influenza virus types.

**Methods:**

An influenza ABCD tetraplex real‐time RT‐PCR (RT‐qPCR) was developed in the frame of this study. A selection of reference virus strains and more than 4000 porcine samples from a passive IAV surveillance programme in European swine with acute respiratory disease were examined.

**Results:**

Two IBV, a single IDV but no ICV infections were identified by tetraplex RT‐qPCR. IBV and IDV results were confirmed by conventional RT‐PCR and partial sequence analysis.

**Conclusions:**

The tetraplex RT‐qPCR proved fit for purpose as a sensitive, specific and high‐throughput tool to study influenza virus transmission at the human‐animal interface. Complementing close‐meshed active virological and serological surveillance is required to better understand the true incidence and prevalence of influenza virus type B, C and D infections in swine.

## 1 INTRODUCTION

Infections with influenza A virus (IAV) are widespread among domestic swine populations worldwide. Due to acute respiratory diseases caused by IAV, clinically often enhanced by further viral and bacterial co‐infections, the pork‐producing industry faces substantial economic losses.^1^, ^2^, ^3^

Influenza A virus belongs to the *Orthomyxoviridae* family and has a genome with eight segments. Subtypes are categorized by the genetic and antigenic properties of the hemagglutinin (HA) and neuraminidase (NA) membrane glycoproteins: 18 HA types (H1‐H18) and 11 NA (N1‐N11) types are distinguished today.^4^, ^5^ Swine are susceptible to different IAV types, of both avian and human origins, and currently perpetuate autonomous porcine‐adapted lineages of subtypes H1, H3, N1 and N2.^6^ In addition, sporadic and dead‐end infections in swine with avian influenza viruses of various subtypes have been reported.^6^ All human pandemic IAVs of the 20th century (with the exception of the 1958 H2N2 viruses) and the current century established autonomously circulating lineages in swine populations following human‐to‐swine transmission.^6^ The most recent human pandemic virus (H1N1/2009, the so‐called swine flu) was likely derived from Mesoamerican swine populations. This emphasizes the zoonotic risk of swine influenza A viruses (SIV).^7^, ^8^ Zoonotic (swine‐to‐human) and reverse zoonotic (human‐to‐swine) transmissions of IAV seem to be occurring regularly.^9^, ^10^, ^11^, ^12^

Aside from IAV, there are two further human influenza virus types, influenza B virus (IBV) and influenza C virus (ICV).^13^, ^14^, ^15^ Similar to IAV, influenza B viruses have eight genome segments including genes encoding HA and NA glycoproteins which, however, have evolved only two distinguishable HA lineages so far.^16^, ^17^, ^18^ Like IAV, IBV is regularly involved in seasonal influenza outbreaks in humans but until to date, only three cases of florid IBV infections have been detected.^14^, ^19^ In Europe, serological studies from the 1960s showed the sporadic presence of antibodies against IBV in domestic pigs in Hungary.^20^ More recently, a surveillance for IBV in U.S. Midwest swine farms showed a seroprevalence of 7.3% at the sample and 38.5% at farm level, respectively.^21^ Furthermore, the susceptibility of swine for IBV has been demonstrated under experimental conditions with pigs developing influenza‐like symptoms as well as lung lesions. The transmission of the virus to sentinel pigs was also seen in this challenge trial.^21^

Influenza C virus is the third human influenza virus type and is composed of seven genome segments. Instead of HA and NA, this virus expresses a hemagglutinin‐esterase‐fusion (HEF) protein on its surface, conveying both receptor‐binding and ‐destroying functions.^22^ It causes only mild diseases in humans, particularly in very young children, and is therefore not included in vaccination schemes.^23^, ^24^ The only report of an infection in swine came from China in 1981, where ICV was found in 15 samples during a year‐long monitoring at a slaughterhouse. Detections were confined to the winter and spring months, in pigs with no signs of illness.^25^

In 2011, a novel C‐like influenza virus has been described in swine in the United States.^26^ Like ICV, it is composed of seven genome segments and presents a HEF protein at the membrane surface. However, the low genetic homology and lack of antibody cross‐reactivity to ICV led to its designation as a new influenza virus type, tentatively named influenza D virus (IDV).^27^, ^28^ To this day, it was found in swine but more frequently in cattle showing mild respiratory disease suggesting cattle may be the domestic reservoir species for IDV.^29^, ^30^, ^31^ No human cases were discovered thus far though antibodies for IDV were found in people with close contact to cattle and swine, and in vitro studies demonstrated the ability of the virus to grow in human‐derived cells.^32^, ^33^, ^34^

In the wake of the H1N1pdm/2009 human pandemic, European surveillance projects for swine IAV revealed that four main IAV lineages were in circulation in domestic swine populations in Europe, one of which was of avian and three of human origin.^35^ Very little is known concerning the occurrence of IBV, ICV and IDV in European swine populations and other animal hosts.^20^, ^21^, ^25^, ^26^, ^27^, ^28^, ^29^, ^30^, ^32^, ^33^, ^34^, ^36^ In the light of the fact that most of the porcine IAV strains established in swine are of human origin, it is conceivable that this could also be possible for human IBV and ICV.^2^, ^3^, ^10^, ^11^, ^35^ Similar to IBV and ICV, there is no systematic monitoring of IDV in swine, although a few reports carried out in European countries revealed anecdotical presence of IDV in Luxemburg and Italy in swine with influenza‐like disease.^31^, ^32^, ^36^

For a better understanding of the epidemiology of IBV, ICV and IDV in swine, a time‐efficient and low‐cost diagnostic tool for large‐scale screening of four influenza virus types was developed here. The influenza ABCD tetraplex reverse transcription real‐time PCR (RT‐qPCR) generically targeting IAV, IBV, ICV and IDV was validated and used to screen 4033 samples from pigs with respiratory disease obtained from 707 farms in twelve European countries.

## 2 MATERIALS AND METHODS

### 2.1 Field samples, reference viruses

**Figure 1:**
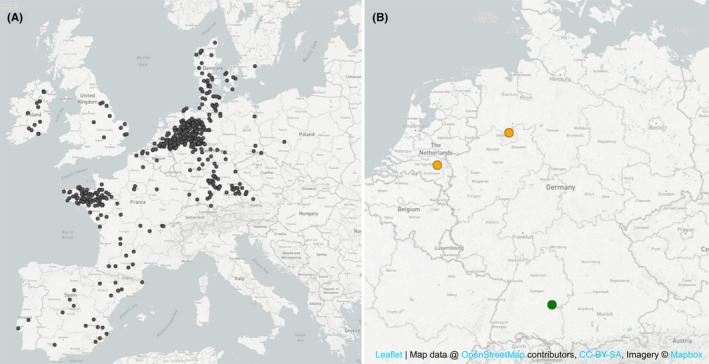
Collection sites of the field samples. Maps were calculated based on latitude and longitude data with the online tool Microreact (http://www.edge.microreact.org/).^41^ Part A shows all collection sites distributed over Middle Europe, and Part B shows the location of the positive samples (orange: IBV; green: IDV)

A industrial‐funded passive surveillance project for IAV infections in swine targeting 12 different European countries, intended as a follow‐up to the European Surveillance Network for Influenza in Pigs (ESNIP) surveillance programme (Ref. 37, Henritzi et al., in preparation), was carried out between April 2015 and March 2017. Field samples obtained from 707 farms were taken from domestic pigs of all ages with signs of respiratory illness. In total, 4033 clinical samples, nasal swabs (n = 3963), lung tissues (n = 46), oral fluids (n = 23) and one lung lavage were obtained. Table 1A and B provides detailed accounts of sample origin. The majority of submissions came from Germany, France and the Netherlands, although the surveillance in Germany started not before July 2016 (Table 1A). Collection sites are illustrated in Figure 1A. Samples were processed and analysed for IAV‐RNA by an M‐gene‐specific RT‐qPCR that was modified after 2009 to include the detection of the pandemic H1N1/2009.^38^ Additionally, the use of an internal control was added to ensure integrity of extracted RNA and inhibitory effects on PCR. Viral RNA of 3862 IAV‐negative samples and 171 IAV‐positive samples stored at −80°C following examination for IAV for up to two years was examined in batches for influenza virus types B, C and D, and re‐examined for influenza A virus. Reference IAVs, IBVs, ICVs and IDVs of swine and other host species were retrieved from the reference collections at Friedrich‐Loeffler‐Institut, Greifswald‐Insel Riems, Germany, the Robert‐Koch‐Institut, Berlin, Germany (Dr. B. Schweiger), Department of Molecular Biology, University of Salzburg (Dr. R. Vlasak), and Istituto Zooprofilattico Sperimentale della Lombardia e dell’ Emilia Romagna, Italy (Dr. E. Foni), and used for RT‐qPCR validation purposes.

**Table 1:** Number of field samples collected from European domestic swine. IAV‐negative samples were collected from April 2015 until March 2017 from twelve European countries (A). Samples from IAV‐positive farms were taken in October to February in 2015, 2016 and 2017, and all farms were positive for IAV, but not all samples were tested positive (B)

(A)								
Countries	Total		2015		2016		2017	
	Samples	Farms	Samples	Farms	Samples	Farms	Samples	Farms
All	3,753	682	631	62	2,391	442	735	180
DE	1,000	386	—	—	688	253	312	133
FR	1,166	122	312	28	703	77	151	17
NL	726	82	138	16	524	57	64	9
DK	282	27	51	3	152	16	79	8
ES	198	21	52	6	117	12	29	3
BE	109	16	22	4	77	11	10	1
UK	156	15	46	4	60	6	50	5
IRL	80	8	—	—	60	6	20	2
PT	14	2	10	1	4	1	—	—
AT	2	1	—	—	2	1	—	—
PL	10	1	—	—	—	—	10	1
SE	10	1	—	—	—	—	10	1

(B)					
Month	Countries	Farms	Samples	IAV‐positive samples	IAV‐negative samples
All	BE, DE, DK, ES, FR, IRL, NL, UK	25	280	171	109
October 2015	FR, UK	2	22	14	8
November 2015	BE, DK, FR	4	62	55	7
February 2016	ES, NL	4	68	39	29
October 2016	DE, FR, IRL, NL	6	62	37	25
November 2016	DE	2	5	4	1
January 2017	DE, DK, ES	7	61	22	39

### 2.2 Design of primers and probes

Primers and probes for detection of IAV, IBV, ICV and IDV for use in a tetraplex RT‐qPCR were selected from previously published assays [M1.2: Hoffmann et al^38^; IBV_HA: Hopkins et al^39^ modified; panIDV_HEF_fwd: Ducatez et al^36^]. In addition, further primers and probes were designed based on alignments comprising full‐length IBV‐NP, ICV‐M, ICV‐HEF, IDV‐NP and IDV‐HEF sequences extracted from the National Center for Biotechnology Information (NCBI) GenBank database (https://www.ncbi.nlm.nih.gov/genbank/), Influenza Research Database (IRD) (https://www.fludb.org/) and the Global Initiative on Sharing All Influenza Data (GISAID) EpiFlu^™^ Database (http://platform.gisaid.org/). Melting temperatures and basic properties of oligonucleotides were approximated using the online tool “Oligocalc”.^40^ Primer and probe sets (Table 2A) were evaluated using strains of the reference collections (Table 3). Based on these results and on comparisons with published assays, further rounds of optimizing oligonucleotide sequences were initiated. Table 2A,B lists final sets of primers and probes for RT‐qPCRs (A) and conventional RT‐PCRs (B).

**Table 2:** Attributes of primers and probes employed in the ABCD tetraplex RT‐qPCR (A) or in conventional uniplex RT‐PCRs (B) for the detection of IAV, IBV, ICV and IDV

(A)						
Primer/Probe	Concentration	Sequence, labelling	Location	Product size	Reference sequence	Comment
IAV_M1.2_M						
IAV‐M1‐F	10 pmol/rxn	agatgagtcttctaaccgaggtcg	(‐2)‐22	101 bp	A/Regensburg/D6/2009	Hoffmann et al^38^
IAV‐M1.1‐R	15 pmol/rxn	tgcaaaaacatcttcaagtytctg	76‐99		(H1N1pdm) [M]	modified
IAV‐M1.2‐R	15 pmol/rxn	tgcaaagacactttccagtctctg	76‐99		FN401576	
IAV‐M1‐FAM	1,25 pmol/rxn	FAM‐tcaggccccctcaaagccga‐BHQ1	49‐68			
IBV_HA						
IfB‐F	10 pmol/rxn	aaatacggtggattaaataaaagcaa	940‐965	170 bp	B/Brisbane/60/2008 [HA]	Hopkins et al^39^
IfB‐R	10 pmol/rxn	ccagcaatagctccgaagaaa	1089‐1109		CY115151	modified
IfB_P	1,25 pmol/rxn	ROX‐cacccatattgggcaatttcctatggc‐BHQ2	994‐1020			
ICV_M						
FluC‐F	10 pmol/rxn	cataattgaacttgtcaatggt	921‐942	94 bp	C/JJ/1950 [M]	
FluC‐R	10 pmol/rxn	catcgagtcaatttcaggca	995‐1014		FR671424	
FluC‐P	1,25 pmol/rxn	Cy5‐tccacaccatctctcccatctgcc‐BHQ2	952‐975			
IDV_NP						
D_NP_F	10 pmol/rxn	cttgaaaagattgcaaatgcag	220‐241	99 bp	D/swine/Italy/199724‐3/2015 [NP]	
D_NP_R	10 pmol/rxn	gttgggtttcagtgccattc	299‐318		KT592534	
D_NP_SO	1,25 pmol/rxn	Hex‐cactacatttcccagctgttgactcc‐BHQ1	264‐289			

(B)						
Primer	Concentration	Sequence, labelling	Location	Product size	Reference sequence	Comment
panIBV_NP						
IBV_SIV_F	5 pmol/rxn	agaugaaugaugucuguuucc	608‐628	340 bp	B/Brisbane/60/2008 [NP]	
IBV_SIV_R	5 pmol/rxn	agggttagrtcttcaatgtc	928‐947		CY115154	
panICV_HEF						
ICV_SIV_F	5 pmol/rxn	aacugauaccacuguaacc	1305‐1323	252 bp	C/JJ/1950 [HEF]	
ICV_SIV_R	5 pmol/rxn	tcatgagaaatyctgtcattc	1557‐1577		FR671422	
panIDV_HEF						
FluD_HE‐1267F	5 pmol/rxn	cccaagtatggcagatg	1243‐1259	266 bp	D/swine/Italy/199724‐3/2015 [HEF]	Ducatez et al^36^
IDV_SIV_R	5 pmol/rxn	ttgtcaaatccagcctgc	1515‐1532		KT592533	

rxn—volume reaction of 25 μL.

**Table 3:** Analytical specificity of primers and probes for detection and discrimination of influenza virus types A, B, C and D. Results are based on the ABCD tetraplex RT‐qPCR

Strain	Subtype/Lineage		RT‐qPCR [cq‐values]				PCR target	Accession number
			IAV	IBV	ICV	IDV		
A/Fort Monmoth/1/1947	H1	N1	16.74	neg	neg	neg	MP	EPI240880, CY045781
A/Wild duck/Germany/WV30/2006	H1	N1	18.01	neg	neg	neg	MP	EPI248511
A/White‐fronted goose/Germany‐NI/R482/2009	H1	N1	18.3	neg	neg	neg	MP	no MP Seq. [HA EPI248525]
A/Germany/Regensburg/2009	H1pdm	N1pdm	19.54	neg	neg	neg	MP	FJ970928
A/Germany‐MV/R26/2011	H1pdm	N1pdm	18.04	neg	neg	neg	MP	EPI356425
A/Swine/Belzig/2001	H1av	N1av	14.26	neg	neg	neg	MP	EPI236901, DQ102484
A/Swine/Germany/R819/2010	H1av	N1av	15.99	neg	neg	neg	MP	EPI411926
A/Swine/Germany/R1738/2010	H1av	N1av	17.66	neg	neg	neg	MP	no MP Seq. [HA EPI411955]
A/swine/Germany‐NI/R369/09	H1 av	N2	19.37	neg	neg	neg	MP	EPI411878
A/swine/Bakum/1832/2000	H1hu	N2	17.41	neg	neg	neg	MP	EPI99278, DQ186977
A/swine/Germany‐NI/R757/10	H1hu	N2	15.44	neg	neg	neg	MP	EPI411916
A/Swine/Germany‐NI/R3394/2009	H1hu	N1av	16.01	neg	neg	neg	MP	EPI411892
A/Swine/Germany/R75/2011	H1pdm	N2	14.61	neg	neg	neg	MP	EPI356461
A/Swine/Germany‐NW/R708/2010	H1pdm	N1av	18.54	neg	neg	neg	MP	EPI301660
A/Swine/Bakum/909/1993	H3	N2	19.03	neg	neg	neg	MP	EPI174502, EU478801
A/Swine/Germany/R96/2011	H3	N2	14.48	neg	neg	neg	MP	no MP Seq. [HA EPI411978]
A/Swine/Germany/R76/2011	H3	N2	17.77	neg	neg	neg	MP	no MP Seq. [HA EPI411965]
B/Beijing/1/94	B	Yamagata	neg	14.66	neg	neg	HA	EPI4555, AF059988
B/Jiangsu/10/2003	B	Yamagata	neg	16.3	neg	neg	HA	EPI159946, CY033844
B/Massachusetts/02/2012	B	Yamagata	neg	13.86	neg	neg	HA	KC892118
B/Malaysia/2506/2004	B	Victoria	neg	16.58	neg	neg	HA	EPI175755, CY038287
B/Brisbane/60/2008	B	Victoria	neg	28.37	neg	neg	HA	EPI173277, FJ766840
C/JJ/1950	C		neg	neg	21.09	neg	MP	EPI283751, FR671424
C/JHB/1/66	C		neg	neg	23.89	neg	MP	no MP Seq. [HE EPI230654, AY880247]
C/Johannesburg/4/67	C		neg	neg	22.99	neg	MP	EPI816669, LC123816
C/NewJersey/76	C		neg	neg	21.26	neg	MP	EPI231872, AB099600
C/Greece/79	C		neg	neg	21.77	neg	MP	EPI231604, AB099602
C/Yamagata/3/2000	C		neg	neg	21	neg	MP	EPI231953, AB099582
C/Miyagi/4/2002	C		neg	neg	23.43	neg	MP	EPI816678, LC123825
C/Yamagata/15/2004	C		neg	neg	20.93	neg	MP	EPI816685, LC123832
C/Yamagata/3/2005	C		neg	neg	22.22	neg	MP	EPI816695, LC123842
C/Yamagata/1/2007	C		neg	neg	22.1	neg	MP	EPI816706 LC123853
C/Yamagata/2/2010	C		neg	neg	22.01	neg	MP	EPI816712, LC123859
D/swine/Italy/199724‐3/2015	D		neg	neg	neg	26.59	NP	KT592534
D/swine/Oklahoma/1334/2011	D		neg	neg	neg	29.74	NP	JQ922309
Tetraplex mixture			FAM	ROX	Cy5	HEX		

### 2.3 One‐step RT‐qPCR

The AG‐Path‐ID^™^ One‐Step RT‐PCR Kit (Ambion) was used throughout. Thermocycling conditions on a Bio‐Rad CFX96 real‐time PCR detection system were optimized by adapting annealing time and temperature. These cycling conditions were found to be optimal for the generic ABCD‐specific RT‐qPCR:

- 10 minutes 45°C, 10 minutes 95°C, 42 cycles each of 15 seconds 95°C ‐ 20 seconds 55°C ‐ 30 seconds 72°C.

### 2.4 Copy‐based standard for ABCD‐Flu‐specific RT‐qPCRs

For the production of RNA run‐off transcripts, products of the above‐mentioned RT‐qPCRs were generated from the IAV strain A/Regensburg/D6/2009 (M gene), the IBV strain B/Brisbane/60/2008 (HA gene), ICV strain C/JJ/1950 (M gene) and IDV strain D/sw/Italy/199724‐3/2015 (NP gene). Products of the correct size were cloned into the pCR^®^II‐plasmid (Invitrogen) containing T7‐ and Sp6‐promoter sequences following the recommendations of the Topo TA cloning Dual Promoter Kit (Invitrogen, Carlsbad, CA, USA). Inserts of selected clones were sequenced using SP6 and T7 primers. RNA was transcribed with T7 RNA polymerase (Promega, Mannheim, Germany) from plasmids linearized with *Hind*III (NEB, Frankfurt, Germany) according to the manufacturer's instructions. Transcribed RNA was further purified by the RNeasy Mini Kit (Qiagen, Hilden, Germany) and quantified using the NanoDrop ND‐1000 Spectrophotometer (PEQLAB Biotechnologies, Erlangen, Germany). Calculation of the RNA copy number was done by the online tool Endmemo (www.endmemo.com/bio/dnacopynum.php/). Triplicate serial 10‐fold dilutions of the purified transcribed RNAs starting at 10^8^ copies/reaction down to one copy were used in the ABCD tetraplex RT‐qPCR to determine the limit of detection (LOD). Samples with a Cq‐value below 40 were considered positive.

### 2.5 Conventional one‐step RT‐PCR

The Superscript III One‐Step RT‐PCR Kit with Platinum Taq polymerase (Invitrogen) was used for conventional RT‐PCR. Thermocycling conditions on an Analytik Jena Flex Cycler or a SensoQuest Labcycler were optimized to the following conditions for generic panIBV_NP, panICV_HEF and panIDV_HEF RT‐PCRs:

- 30 minutes 48°C, 2 minutes 94°C, 45 cycles each of 30 seconds 94°C ‐ 30 seconds 55°C ‐ 40 seconds 68°C, final elongation 2 minutes 68°C.

### 2.6 Sequencing

IBV, ICV or IDV PCR‐positive field samples were analysed by Sanger sequencing of the IBV‐NP, ICV‐HEF, IDV‐HEF gene fragments obtained with the panIBV_NP (340 bp), panICV_HEF (250 bp) and panIDV_HEF (260 bp) conventional RT‐PCRs (Table 2B). Specific amplicons were purified from 1.5% agarose gels using a QIAquick Gel Extraction Kit (Qiagen) and Sanger‐sequenced using the RT‐PCR primers. The sequences were analysed on an ABI 310 sequencer, curated using the Chromas Lite^®^ software (http://www.technelysium.com.au/Chromas250Setup.exe) and assembled using the Geneious^®^10.2.3 software suite (Biomatters Ltd., Auckland, New Zealand).

### 2.7 Molecular sequence analyses

The IRD (https://www.fludb.org/) or GISAID EpiFlu^™^ (http://platform.gisaid.org/) Databases were screened with the BLASTN2 algorithm to identify closely related sequences of the panIBV_NP, panICV_HEF and panIDV_HEF sequences.

### 2.8 Virus isolation on cell culture

Madin‐Darby canine kidney 2 (MDCK‐2, ATCC^®^ CRL‐2936^™^) grew in minimum essential medium (MEM) with 5% foetal calf serum (FCS) in 25‐cm^2^ culture flasks (Corning, Nuembrecht, Germany). For isolation of the positive field samples, medium was replaced with infectious material and incubated for 1 hour at 37°C. After incubation, MEM with 6‐ (1‐tosylamido‐2‐phenyl) ethyl chloromethyl ketone (TPCK)‐treated trypsin was added. After 72 hours, supernatants of the cultures were passaged once after a freeze‐thaw‐step, regardless of a recognizable cytopathic effect (CPE).

### 2.9 Mapping of farm locations

Maps of the collection sites of the field samples were calculated based on latitude and longitude data with the online tool Microreact (http://www.edge.microreact.org/).^41^

## 3 RESULTS

### 3.1 Assembly and analytical performance of an influenza virus type‐specific ABCD tetraplex RT‐qPCR for the simultaneous detection and differentiation of IAV, IBV, ICV and IDV in porcine samples

Various primer/probe sets, either published or newly developed, were tested in monoplex RT‐qPCRs for their sensitivity and specificity using RNA extracted from reference isolates (data not shown). A panel of RNA containing 36 influenza virus strains were used to evaluate different sets of primers and probes for their analytical specificity for four different influenza lineages (Table 3): 17 IAVs of subtypes H1, H3, N1 and N2 of human, avian or porcine origin; five IBVs of the Yamagata and Victoria lineages of human origin; and eleven ICVs of human origin and two IDVs of porcine origin. Oligonucleotide sets were further selected for a combined ABCD tetraplex RT‐qPCR on the basis that no intersubtypic cross‐reactivity occurred and inhibitory interferences were excluded. The final selection comprised previously published but slightly modified sets for the detection of IAV and IBV,^38^, ^39^, ^42^, ^43^ and newly developed sets for the detection of ICV and IDV (Table 2A,B). This set fulfilled the mentioned requirements as shown in Table 3. Non‐specific reactivity of these PCRs with other porcine viral or bacterial respiratory pathogens as listed in Supplementary Table 1 was excluded as well.

**Figure 2:**
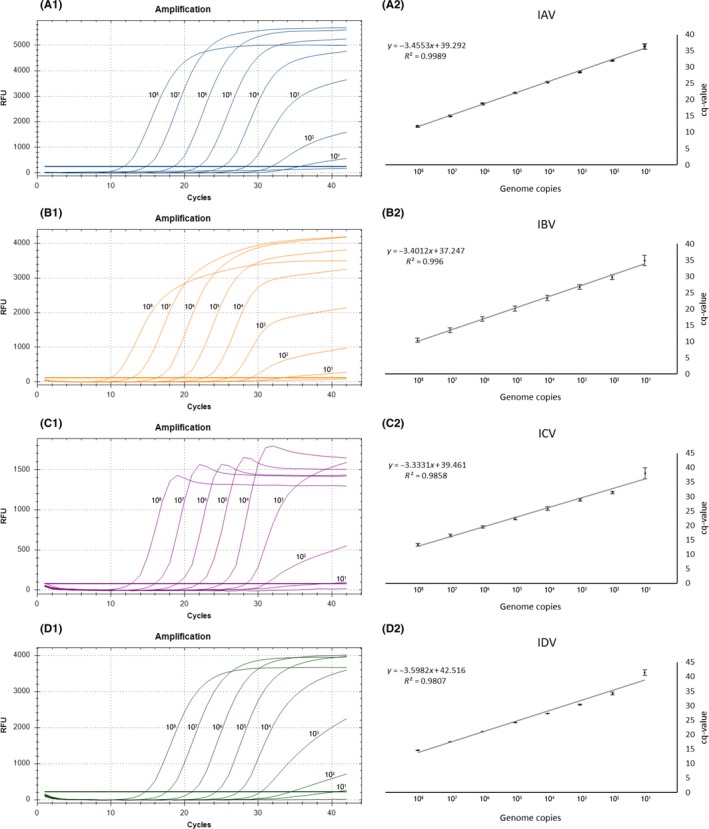
Analysis of the detection limit of the ABCD tetraplex RT‐qPCR based on the mean value of triplicate serial 10‐fold dilutions of transcribed viral RNA ranging from 10^8^ to 1 copies/reaction (A1‐D1). Linear regression analysis revealed correlation coefficients (R2) and slope values (A2‐D2). A, IAV, FAM signal; B, IBV, ROX signal; C, ICV, Cy5 signal; D, IDV, HEX signal

Copy‐based standards were used to determine the limit of detection of the tetraplex RT‐qPCR (Figure 2). An LOD of 10 target copies was evident for all four targets when only a single of the four targets was present.

**Figure 3:**
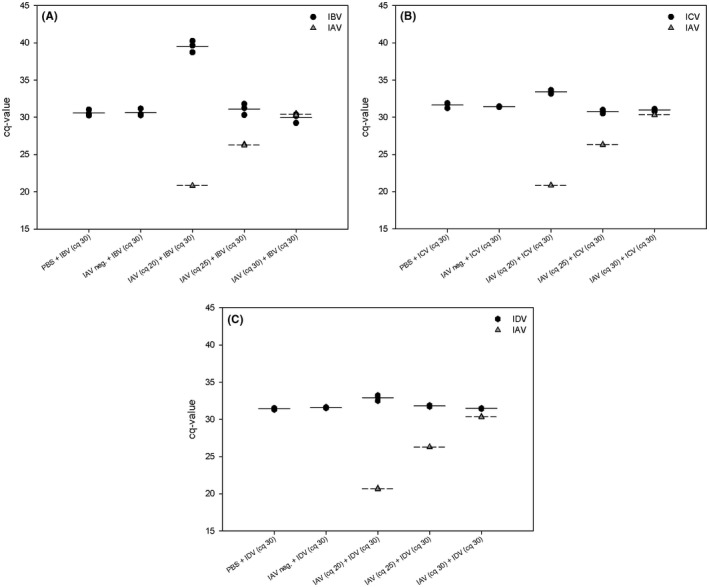
Analysis of the sensitivity of the ABCD tetraplex RT‐qPCR in simulated co‐infections. IAV‐positive swine nasal samples with cq‐values of 20, 25 and 30, and IAV‐negative swine samples and PBS were mixed with RNA of either IBV (A), ICV (B) or IDV (C) each of Cq = 30. Mixtures were analysed three times in the RT‐qPCR

To prove the sensitivity of the tetraplex RT‐qPCR for potential mixed infections of IAV with either IBV, ICV or IDV, IAV‐positive swine nasal samples with initial cq‐values of 20, 25 and 30 were spiked with IBV‐, ICV‐ or IDV‐RNA at a cq‐value of 30. Additional mock samples (PBS) and IAV‐negative swine nasal samples were likewise mixed with IBV‐, ICV‐ or IDV‐RNA as indicated above. Samples were tested three times with the ABCD tetraplex RT‐qPCR. The majority of the B/C/D‐spiked samples yielded results in the frame of the original cq‐value of 30, with the exception of the mixes IAV‐cq 20 + IBV‐cq 30 where IBC cq‐values close to the upper detection threshold of cq 40 resulted (Figures 3A,B, Table S2). In reverse fashion, further mixtures of IBV, ICV and IDV with initial cq‐values of 20, 25 and 30 were spiked with IAV at cq 30 and were tested . In addition, triple and 4‐fold mixtures were examined as well. All yielded results close to the initial cq‐values (Table S2).

### 3.2 Diagnostic performance of the influenza virus type‐specific ABCD tetraplex RT‐qPCRs

#### 3.2.1 Screening for IBV, ICV and IDV in European domestic swine populations April 2015‐March 2017

Only three IAV‐negative samples from three different farms yielded a positive RT‐qPCR result for either IBV or IDV, albeit with high cq‐values, (Table 4, Figure 1B). To exclude the possibility of false‐positive results due to sample or PCR contamination, re‐extracted RNA of these samples was examined in conventional IBV‐ or IDV‐specific RT‐PCRs as listed in Table 1B. Sequence analysis of these amplicons confirmed the findings of the ABCD tetraplex RT‐qPCR (sequences listed in Table S3). The two confirmed IBV‐positive cases were detected in samples from the Netherlands (age of swine unknown) and Germany (adult sow), respectively. Temporally, the detection was made in samples collected between May and September, which is well outside the human influenza season in these countries. The IDV case was found in a swine of unknown age in Germany. No ICV‐positive porcine samples were detected. Expectedly, no IAV‐positive results were obtained as a pre‐selection of IAV‐negative samples was used here.

**Table 4:** Field samples positive for IBV and IDV. Results are based on the ABCD tetraplex RT‐qPCR and conventional uniplex RT‐PCR

Sample^a^	Result	Country	Collection month	Specimen	Swine category	RT‐qPCR [cq‐value]	RT‐PCR	Closely related virus [identity]
AR 3087/16	IBV	NL	May 2016	Nasal swab	No data	35.73 (IBV)	+ (IBV)	B/Santa Cruz/194/2012; NP [281/283 (99%)]
AR 6877/16	IBV	DE	September 2016	Nasal swab	Sow	37.76 (IBV)	+ (IBV)	B/New York/1231/2009; NP [297/299(99%)]
AR 4484/16	IDV	DE	July 2016	Nasal swab	No data	34.24 (IDV)	+ (IDV)	D/swine/Oklahoma/1334/2011; HEF [188/196 (95%)]

a Sample indicates the identity of a sample (AR NNNN) and the year of sampling (/YR).

Madin‐Darby canine kidney cell culture isolation of the two IBV‐ and the IDV‐positive samples was unsuccessful ruling out further analysis. Serological studies of the farms which tested positive for IBV and IDV could not been carried out, since serum samples were not available and the animals no longer retrievable on the farms.

**Figure 4:**
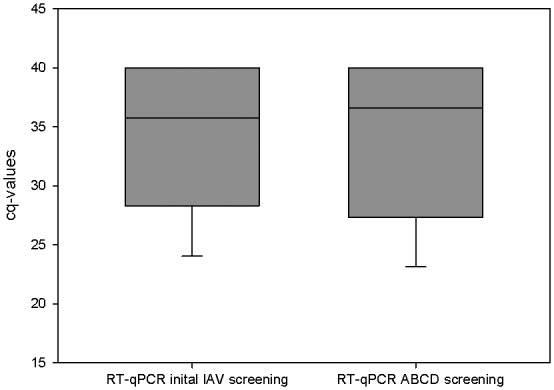
Comparison of the IAV cq‐values from the initial (monoplex‐) IAV screening and the cq‐values of the IAV (BCD)‐tetraplex RT‐qPCR. First screening was done within a week of sample receipt; thereafter, RNA was stored at −80°C up to 26 months. Cut‐off for the ABCD tetraplex RT‐qPCR was set at cq 40

To detect possible co‐infection of IAV with any of the three other influenza virus types, 171 IAV‐positive samples from 25 farms of eight European countries were analysed in addition (Table 1B). The cq‐values initially obtained by monoplex IAV screening when the samples were submitted and the IAV cq‐value obtained by ABCD tetraplex RT‐qPCR after storage of RNA for up to 26 months at −80°C differed only slightly, as shown in Figure 4. This indicated stability over time of the RNAs analysed. None of these 171 IAV‐positive porcine samples yielded IBV, ICV or IDV.

## 4 DISCUSSION

The zoonotic propensity of porcine IAV is well established and has been demonstrated impressively during the human H1N1 pandemic of 2009.^2^, ^35^ In this respect, it is astonishing to note that, in Europe, after the discontinuation of the ESNIP3 monitoring programme,^37^ so far only France runs a sustained government‐administrated surveillance programme targeting IAV in swine populations.^44^, ^45^, ^46^ No efforts have been undertaken to gather knowledge on putative IBV and ICV infections in European swine although sporadic incidents had been reported in the past.^20^, ^21^, ^25^ IDV likewise was not a target of investigation programmes, with the exception of some IDV screening in Italian swine, showing a virus prevalence of 2% and a seroprevalence of 11.7%, and serological studies in Luxembourgian swine showing an increasing seroprevalence from 0 to 5.9% in the years 2012‐2015.^31^, ^32^, ^36^

To provide a cost‐effective tool that enables, in standard molecular diagnostic laboratories, the simultaneous and high‐throughput screening of four influenza virus types in porcine samples, we successfully have developed an ABCD tetraplex RT‐qPCR. Analytical and diagnostic performance characteristics proved this PCR fit for purpose.

An ongoing, industrial‐funded passive surveillance project for porcine IAV intended as a follow‐up of the ESNIP3 programme in Europe provided samples from pigs with respiratory disease. The primary interest of the present study, however, was not on IAV but focused on IBV, ICV and IDV infections. Thus, a pre‐selection of IAV‐negative samples from swine with respiratory disease was used which comprised 3,753 samples from 682 farms collected in twelve European countries. These samples yielded only two swabs that were positive with low virus loads for IBV (one sample each from the Netherlands and from Germany), and IDV was detected in only one German sample, no case of ICV was seen. Nevertheless, evidence of an IBV infection was confirmed for the first time in European swine and an IDV infection was not previously reported from pigs in Germany. It is of interest to note that the IBV detections occurred during the summer period when no human influenza activity is registered in Europe. Thus, it remains unclear whether the IBV detected here in swine originated from a temporally closely associated human‐to‐swine transmission. Considering that no IBV reservoir in animals has been identified so far, this would suggest limited but autonomous replication of IBV in some swine populations. Unfortunately, it was not possible to trace sources and spread of the IBV and IDV infections in the respective swine holdings. Narrow‐meshed, regionally focused virological as well as serological surveillance including also healthy pigs would be required to clarify the actual prevalence of IBV and IDV in swine populations.

Information on possible co‐infections with IAV and the other influenza virus types in swine is not available; in humans, co‐infection of IAV/IBV was seen in 1.6% of a case study.^47^ This aspect was addressed by examining an additional 171 samples from 25 farms with swine acutely infected with IAV. However, none were positive for IBV, ICV or IDV. These samples were obtained during the human flu season in 2016 and 2017 (January‐April), when low IBV activity was confirmed in humans.

Samples analysed in this study were not representative for the swine populations in the different European countries; it is therefore not possible to draw conclusions regarding the true incidence of influenza virus infections. Also, no data from healthy swine have been collected, since the samples originated from passive surveillance targeting only swine with acute respiratory disease; a similar knowledge gap also exists for IAV infections in healthy pigs. A broader approach by an active surveillance for influenza virus types would be required to better understand endemic circulation at least of IAV in larger swine holdings. The newly developed tetraplex RT‐qPCR is an appropriate tool for this purpose which may also cast further light on potential reverse zoonotic transmission events of IBV and ICV and the presence of IDV in swine populations. In addition, the ABCD tetraplex RT‐qPCR should also be useful in screening samples of other host species including man. Therefore, projects aiming at the examination of influenza virus transmission at the swine‐human interface might benefit from this tool.
